# The utility of low-density genotyping for imputation in the Thoroughbred horse

**DOI:** 10.1186/1297-9686-46-9

**Published:** 2014-02-04

**Authors:** Laura J Corbin, Andreas Kranis, Sarah C Blott, June E Swinburne, Mark Vaudin, Stephen C Bishop, John A Woolliams

**Affiliations:** 1 Roslin Institute and Royal (Dick) School of Veterinary Studies, University of Edinburgh, Easter Bush, Midlothian EH25 9RG, UK; 2 Aviagen Ltd, Newbridge, Midlothian EH28 8SZ, UK; 3 Animal Health Trust, Newmarket CB8 7UU, UK

## Abstract

**Background:**

Despite the dramatic reduction in the cost of high-density genotyping that has occurred over the last decade, it remains one of the limiting factors for obtaining the large datasets required for genomic studies of disease in the horse. In this study, we investigated the potential for low-density genotyping and subsequent imputation to address this problem.

**Results:**

Using the haplotype phasing and imputation program, BEAGLE, it is possible to impute genotypes from low- to high-density (50K) in the Thoroughbred horse with reasonable to high accuracy. Analysis of the sources of variation in imputation accuracy revealed dependence both on the minor allele frequency of the single nucleotide polymorphisms (SNPs) being imputed and on the underlying linkage disequilibrium structure. Whereas equidistant spacing of the SNPs on the low-density panel worked well, optimising SNP selection to increase their minor allele frequency was advantageous, even when the panel was subsequently used in a population of different geographical origin. Replacing base pair position with linkage disequilibrium map distance reduced the variation in imputation accuracy across SNPs. Whereas a 1K SNP panel was generally sufficient to ensure that more than 80% of genotypes were correctly imputed, other studies suggest that a 2K to 3K panel is more efficient to minimize the subsequent loss of accuracy in genomic prediction analyses. The relationship between accuracy and genotyping costs for the different low-density panels, suggests that a 2K SNP panel would represent good value for money.

**Conclusions:**

Low-density genotyping with a 2K SNP panel followed by imputation provides a compromise between cost and accuracy that could promote more widespread genotyping, and hence the use of genomic information in horses. In addition to offering a low cost alternative to high-density genotyping, imputation provides a means to combine datasets from different genotyping platforms, which is becoming necessary since researchers are starting to use the recently developed equine 70K SNP chip. However, more work is needed to evaluate the impact of between-breed differences on imputation accuracy.

## Background

The introduction of high-throughput, single nucleotide polymorphism (SNP) chips that permit the analysis of large numbers of SNPs in parallel has enabled large-scale studies of human and livestock populations. A common feature of genome-wide association studies (GWAS) is that large sample sizes are needed to ensure sufficient power to detect what are hypothesised to be quantitative trait loci (QTL) with relatively small effects. To validate any detected QTL, both a substantial number of samples for the initial analysis and a second independent sample are required. Furthermore, any underlying data structure, such as that caused by different ancestries, e.g. different breeds in the case of livestock, and the presence of environmental factors, has the potential to reduce power for a given sample size.

In the equine setting, the accumulation of large numbers of samples represents a significant challenge. Since the introduction of the first equine SNP chip by Illumina in 2007, several GWAS of monogenic diseases have been successful in identifying associated regions of the genome and in several cases, causal mutations [1-3]. However, results for the analysis of complex traits have been less convincing; some studies have reported QTL, but many of these QTL have been defined with *ad hoc* significance thresholds, since authors attempt to balance the risk of Type I and Type II errors [4,5]. The apparently low signal to noise ratio is an indication of the low power, caused in part by small sample sizes. Moreover, insufficient validation has been done to confirm whether or not these initial findings are true associations or false positives. One of the reasons for small sample sizes is the cost of genotyping. While the cost of genotyping with SNP chips has fallen during the last few years, the cost relative to potential return remains important, and within some sectors of the equine industry, e.g. the UK sport horse sector, the potential to make significant returns from breeding superior animals is generally limited. Therefore, the development of genomic approaches to breeding in the equine industry requires more cost-effective genotyping.

One opportunity to reduce genotyping costs is the development of low-density genotyping. If a reference population of individuals genotyped at high-density is available, individuals from a test population or selection candidates can be genotyped for a subset of these loci on a low-density panel (LDP), followed by imputation to fill in the ‘missing’ SNP genotypes [6]. Provided the reference population and the test population are genetically similar in origin, population genetic models can use correlations between alleles at neighbouring loci measured in the former to predict unobserved genotypes in the latter [6]. The dependence of imputation accuracy on the SNP density in the LDP means that there will always be a trade-off between the cost of genotyping and the accuracy of imputation. Other factors that affect the accuracy of imputation include levels of linkage disequilibrium (LD) in the population, the degree of similarity between the reference population and the test population and, to some extent, the size of the reference population [7-10].

Efforts to develop improved imputation algorithms have resulted in a wide range of software programs, most of which have evolved from programs written to infer haplotype phase from large-scale genotype data. Commonly used programs include fastPHASE [11], MACH [12], IMPUTE [13], AlphaPhase [14] and BEAGLE [15], and their relative efficacies have been explored under various scenarios [7,15-18]. Whereas some of these imputation methods use linkage analysis to exploit known relationships between individuals, in many cases, knowledge of relationships is not required and population-wide LD between SNPs is used.

Because the imputation method relies on LD between SNPs on the LDP and the remaining SNPs on the high-density panel, the choice of SNPs for the LDP also affects the accuracy of imputation. A significant effort has been devoted to optimising LDP SNP selection and several algorithms have been developed along this vein. Many programs use LD between pairs or groups of markers to select LDP SNPs in a so-called block-free approach, e.g. Tagger [19] or LDSelect [20]. Another common approach is to use haplotype information in a block-based approach, e.g. HapBlock [21], while other more novel algorithms have been developed such as the neighbourhood graph approach of Halldórsson et al. [22] or the multiple linear regression approach of He and Zelikovsky [23]. In situations where LDP SNPs are selected to predict haplotypes, they are commonly referred to as ‘tag SNPs’ (see Halldórsson et al. [24] for a review).

In this study, genotypes from the Illumina Equine SNP50 BeadChip (http://www.illumina.com/documents/products/datasheets/datasheet_equine_snp50.pdf) were used to investigate the accuracy of imputation that can be achieved in Thoroughbred horses, without pedigree information, and using a typical imputation program (BEAGLE). Three methods of LDP SNP selection were tested across six LDP sizes in order to evaluate the impact of various SNP selection criteria that involve both information content and LD of SNPs. The effect of geographical substructure on the accuracy of imputation was also investigated.

## Methods

### Sample collection

The data for this study consisted of 853 Thoroughbred horses originating from the United Kingdom (UK dataset), and 348 Thoroughbred horses from the United States (US dataset). The UK dataset had been the subject of two GWAS, and the US dataset had been the subject of a further GWAS, and each GWAS was structured as a case–control study for one of three diseases. None of the GWAS identified any major QTL for their target disease [25] and so for the purpose of this study the horses were treated as population samples from two geographically distinct regions.

### UK dataset

**Figure 1:**
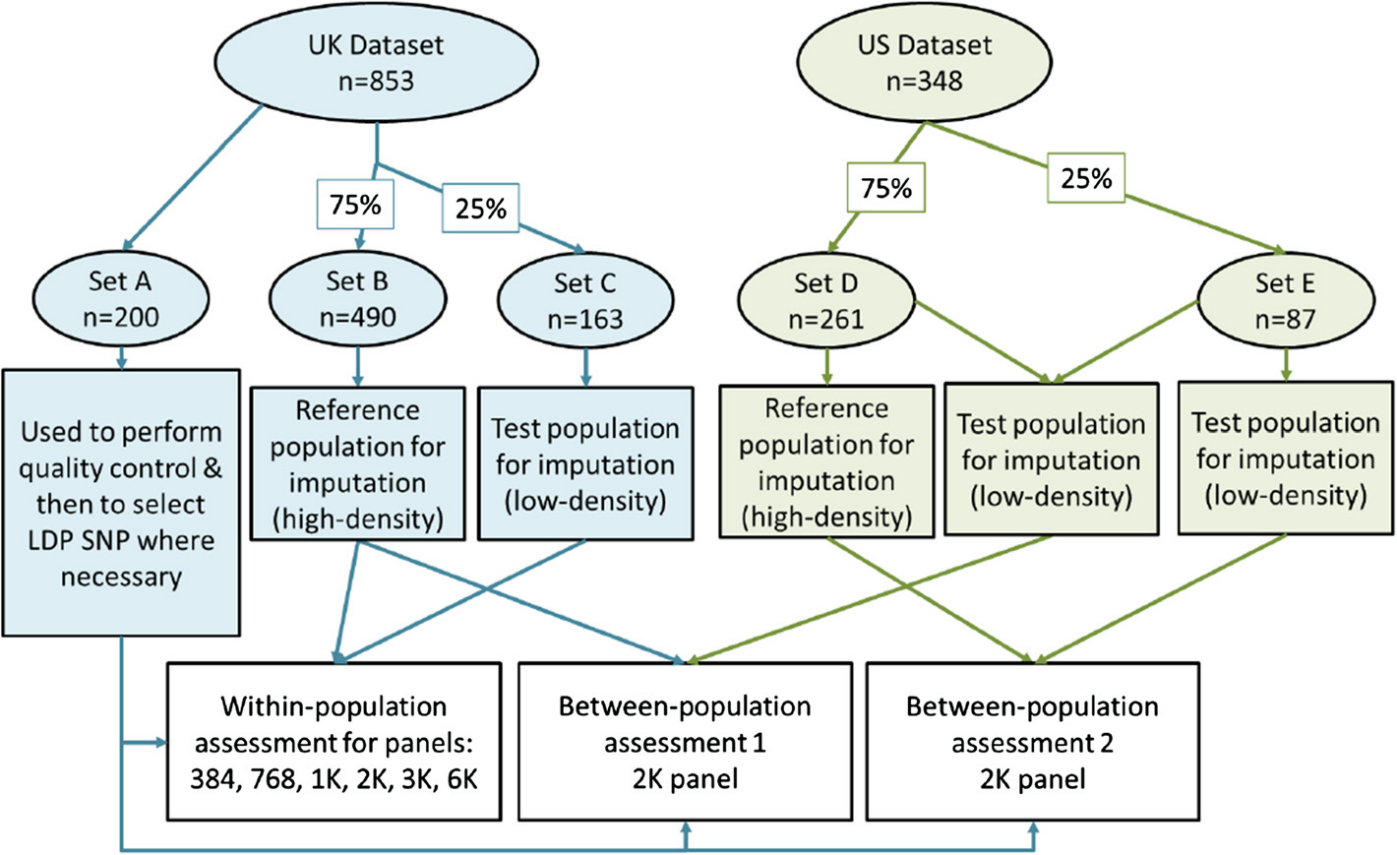
Data flow for analysis.

In the UK, blood samples were collected by the Animal Health Trust between 2006 and 2008, from Thoroughbred horses competing in both flat- and jump-racing (513 males, 340 females). Horses were from a wide geographical area and are expected to be relatively unrelated. Samples in the UK dataset were randomly assigned to one of three subsets: Set A, containing 200 samples, which was used to select LDP SNPs; Set B, containing 490 (75%) of the remaining samples, which was used as the training reference population; and Set C, containing the remaining 163 samples (25%), which was used as the test population, and which were assumed to be genotyped with the LDP. Genotypes for the LDP were obtained by masking SNPs that were not selected to be in the LDP being tested. A graphical representation of this data flow is in Figure 1.

### US dataset

In the US, blood samples were collected over two years (2007 and 2008) from 348 Thoroughbreds (159 males, 189 females) admitted to the Rood and Riddle Equine Hospital, Lexington (Kentucky). Horses originated from one of 19 surrounding horse farms, with the number of horses per farm ranging from two to 89. Since sampling was anonymous, pedigree details for the horses were not available but the data set was expected to consist of a mixture of half-sibs (by sire and by dam since data was collected across two years) and more distantly related horses. Two analyses were performed using this dataset along with the UK dataset to investigate imputation across populations. In the first analysis, the training information obtained from Set B in the UK dataset was used for imputation of the entire US dataset, which was assumed to be genotyped with the LDP. In the second analysis, samples in the US dataset were randomly assigned to one of two subsets: Set D, containing 261 samples (75%), which was used as the training reference population, or Set E, containing the remaining 87 US samples, which was used as the test population and which was assumed to be genotyped with the LDP. A graphical representation of this data flow is in Figure 1.

### Genotyping

All blood samples were collected in EDTA, sent to the Animal Health Trust for further processing, and to Tepnel for DNA extraction (Tepnel has since been bought by Gen-Probe). An aliquot of each sample was diluted to 70 ng/μl and sent to Cambridge Genomic Services (http://www.cgs.path.cam.ac.uk/services/genotyping/) for genotyping using the Illumina Equine SNP50 Genotyping BeadChip, which comprises 54 602 SNPs across all autosomes and the X chromosome. These SNPs were selected from a database of over one million SNPs (http://www.broadinstitute.org/ftp/distribution/horse_snp_release/v2/) generated during the sequencing of the horse genome [26,27]. All samples for this study were genotyped at the same time, along with samples for several other studies. The full dataset, a batch of 1342 samples, was checked using the Illumina GenomeStudio genotyping module. A series of quality control metrics identified 3895 poorly performing SNPs (7.1%) (see Additional file 1: Table S1). Genotypes for these SNPs were set to missing in all samples, leading to their subsequent exclusion during quality control undertaken specifically for this study (see below).

### Quality control

Quality control was applied to Set A in order to generate a list of SNPs that were used in all subsequent stages of the analysis. SNPs that were genotyped in less than 95% of samples in the set and those with a minor allele frequency (MAF) below 0.01 were excluded. The analyses within the UK dataset focused on four *Equus caballus* (ECA) chromosomes: ECA1, ECA10, ECA20 and ECA26; these were chosen to represent the shortest (ECA26), longest (ECA1), and median length (ECA20) chromosomes (measured in cM, based on Swinburne et al. [28]), and to include two centromeric chromosomes (ECA1 and ECA10) and two acrocentric chromosomes (ECA20 and ECA26). In the analyses with the US dataset, only ECA1 and ECA26 were analysed. After quality control, the dataset consisted of 3581 SNPs on ECA1 (18.0% excluded), 1532 SNPs on ECA10 (20.0% excluded), 1225 SNPs on ECA20 (17.5% excluded) and 781 SNPs on ECA26 (18.5% excluded).

### Selection of SNPs for the low-density panel

The three methods were used to select LDP SNPs, as detailed below. Genotype data from the 200 UK samples assigned to Set A were used to generate (i) the MAF of the SNPs for the algorithm used in Methods 2 and 3 and (ii) the LD map used in Method 3 (see below). In the within-population analysis of the UK dataset, the methods were tested at six different densities, representing genome-wide panels with 384, 768, 1K, 2K, 3K and 6K SNPs. The equivalent densities, expressed in terms of N_e_ (effective population size) SNPs per Morgan, as described in Solberg et al. [29], were 0.09, 0.18, 0.24, 0.48, 0.72 and 1.44N_e_/Morgan, assuming N_e_ = 150 and a total genome of 27.72 Morgan [28]. In the subsequent analysis with the US dataset to assess the efficacy of imputation across populations, the LDP density tested was 2K, because at this density the within-population accuracy was always greater than 0.8. The number of LDP SNPs to be selected for a given chromosome (*n*_*chr*_) was proportional to the ratio of the length of the chromosome (*len*_*chr*_) to the whole genome (*len*_*genome*_) in terms of physical distance in base pairs (*n*_*chr*_ = *LDP*_size_*len_*chr*_*/len*_*genome*_).

### Method 1: Equidistant in bp (bpEQ)

SNPs for the LDP were selected such that their spacing along the chromosome was approximately equidistant in base pairs. This was achieved by dividing the total base pair length of the chromosome into equally sized segments, the number of segments being equal to the desired number of LDP SNPs for the given LDP density (*n*_*chr*_) minus 1. The closest SNP to each segment boundary was then chosen to be a SNP in the LDP, irrespective of its MAF.

### Method 2: Equidistant in bp and optimised for MAF (bpMAF)

SNPs for the LDP were selected so that their spacing along the chromosome was approximately equidistant in base pairs and their MAF was high. In order to meet both objectives, SNP selection was performed separately for each chromosome using a custom python program that applied a genetic algorithm. The cost function to be minimized included two components: the first component aimed at driving the MAF of the selected SNPs towards 0.5 by applying a penalty equal to ${\left(0.5-\mathrm{MA}F_{\mathrm{SN}P_{i}}\right)}^{2}$ (1) and the second component ensured equal spacing. An ideal distance between SNPs, *d*, was calculated as: $d=\frac{\mathrm{le}n_{\mathrm{chr}}}{n_{\mathrm{chr}}-1}$ and then, the spacing between consecutive SNPs *i* and *i* + 1 in the LDP was forced to approach *d* using the function: ((*S*_*i*_ - *S*_*i* + 1_) - *d*)^2^ (2), where S is the base pair position of the SNP. The set of *n*_*chr*_ SNPs was then derived by iteratively minimizing the following function over all SNPs:

$$\sum_{n}\left[{\left(0.5-\mathrm{MA}F_{\mathrm{SN}P_{i}}\right)}^{2}+{\left(\left(S_{i}-S_{i+1}\right)-d\right)}^{2}\right].$$

In order to ensure good coverage at the telomeres, where recombination events are more frequent and hence accuracy of imputation is expected to be lower, the SNPs from the high-density panel that were closest to the ends of each chromosome were included in the LDP.

### Method 3: Equidistant in LD units and optimised for MAF (lduMAF)

In the absence of a detailed recombination map for the horse, an alternative measure of distance was used as a proxy, such that the assumption of uniformity of LD and recombination along the length of a chromosome could be removed. The proxy used was linkage disequilibrium units (LDU), as calculated using the LDMAP program described and developed by Maniatis et al. [30]. The theory behind the LDMAP program is based on the Malecot equation [31], and is described extensively elsewhere [32]. An LD map for each chromosome was constructed, using the genotypes of samples in Set A (for further details, see Additional file 2). LD map distance has been shown to have a close relationship with linkage maps [33] and recombination rates, at least to the extent that recombination hot spots can be identified [34]. Maps for all chromosomes can be found in Additional file 3: Figure S1.

SNPs for the LDPs were then selected according to the same algorithm used in Method 2, but with SNP locations given in LDU instead of base pair positions. In cases for which SNPs were allocated to the same position in the LD map, a small addition was made to subsequent locus positions (10^-6^) before entering the SNP locations in the LDP SNP selection algorithm such that SNP order remained consistent with the physical map.

### Imputation

The software program BEAGLE (v 3.3.1) [35] was used to impute from low- to high-density markers without pedigree information, since none was available. The default parameters of the program were used throughout and the most likely genotype was taken to be the imputed genotype at masked loci. For comparison, masked loci were also imputed by random sampling of genotypes, conditional on the allele frequencies at the SNPs observed in the reference populations (Set B or Set D). Because no other quality control was carried out in the reference or test populations, at this stage, a small number of SNPs had a MAF below 0.01 and a very small number of SNPs were monomorphic.

Imputation accuracy was evaluated for the three LDP SNP selection methods, the six LDP densities and four chromosomes, and was summarised per SNP and per individual. For each imputed SNP, imputation accuracy was assessed using two measures: (i) the proportion of genotypes for the SNP that were correctly imputed among samples; and (ii) the correlation between the true and imputed genotypic allele counts across all samples (homozygote for allele 1, coded 0; heterozygous, coded 1; homozygous for allele 2, coded 2). The very small number of loci where true or imputed SNP genotypes were monomorphic among samples, were excluded from the correlation calculations. Summary statistics were then calculated across all SNPs. For each individual, imputation accuracy was calculated using the same two measures: (i) the proportion of all of the horses’ genotypes that were correctly imputed; and (ii) the correlation between the true and imputed genotypes across all the horses’ SNPs when coded as above. As before, monomorphic SNPs were excluded from the correlation. Summary statistics were then calculated across all horses.

Finally, an adjustment to the proportion of correctly imputed genotypes by the expected proportion that would be correct from random sampling of alleles was calculated as: $\frac{\mathrm{accuracy}-\mathrm{random}\_\mathrm{accuracy}}{1-\mathrm{random}\_\mathrm{accuracy}}$[7], where *accuracy* is the proportion of correctly imputed genotypes achieved for the SNP and *random_accuracy* is the expected proportion using random imputation. The expected proportion is given by *p*^4^ + 4*p*^2^*q*^2^ + *q*^4^[9], where *p* and *q* are the frequencies of the major and minor alleles of the SNP in the reference population. This statistic adjusts for the fact that SNPs with a low MAF are likely to be imputed with high accuracy by chance alone.

### Linkage disequilibrium

In order to explore possible causes of differences in imputation accuracy across SNPs, PLINK [36,37] was used to calculate LD between pairs of SNPs using the squared correlation based on genotypic allele counts. This is identical to the *r*^*2*^ measure of LD when mating is at random, i.e. assuming genotypic frequencies are in Hardy-Weinberg equilibrium [38]. However, to denote the distinction from the true *r*^*2*^, the term $r_{g}^{2}$ will be used. Values of $r_{g}^{2}$ were calculated between all pairs of SNPs in Set A. Average pairwise $r_{g}^{2}$ were then calculated for SNPs in 1 Mb sliding windows, with 0.5 Mb overlaps.

## Results

### Within-population assessment of imputation accuracy

**Figure 2:**
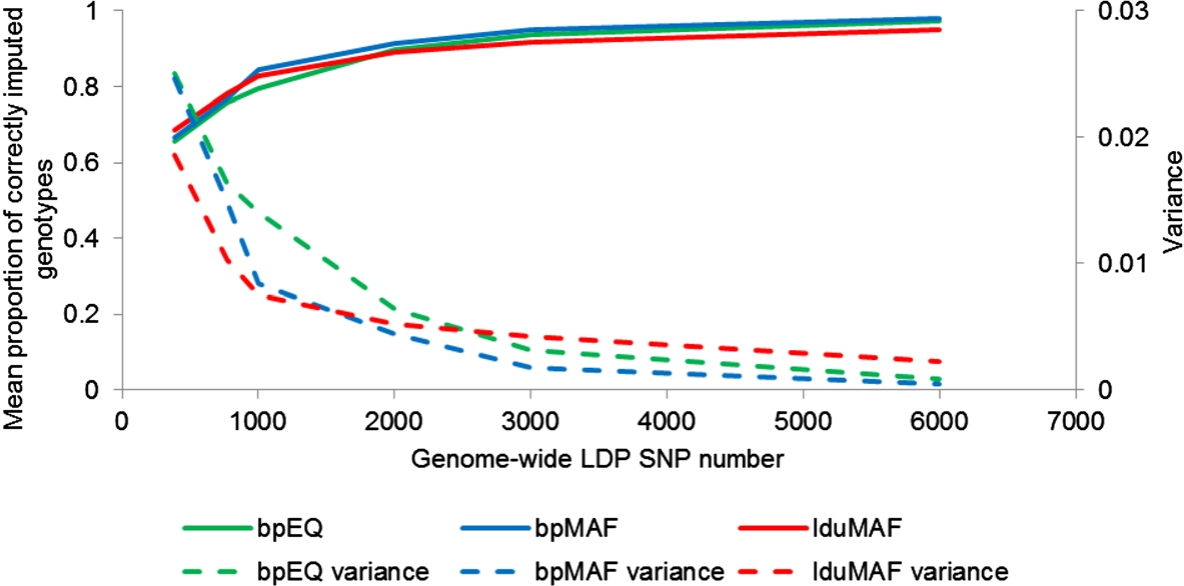
**The mean proportion of correctly imputed genotypes and its variance across SNPs for ECA1.** As calculated in the within-population analysis of the UK dataset and plotted against the total number of SNPs on a genome-wide LDP of equivalent density (figures for all chromosomes are in Additional file 5: Figure S2).

The accuracy of imputation, as measured by the proportion of SNPs correctly imputed, increased as the number of SNPs in the LDP increased, as shown for ECA1 in Table 1 and Figure 2 (and in Additional file 4: Table S2 and Additional file 5: Figure S2, for all chromosomes). For example, using equidistant LDP SNPs (bpEQ), the mean proportion of correctly imputed genotypes ranged from 0.59 at the minimum LDP SNP density of 0.09N_e_/Morgan, to 0.97 at a density of 1.44N_e_/Morgan. The increase was greatest at the lower densities and showed diminishing returns with further increases in density. A large range in the proportion of genotypes correctly imputed was observed between animals, particularly when the density of the LDP was lowest, when the proportion ranged from an average of 0.54 to an average of 0.93 across the three methods for ECA1. Although the difference in accuracy between the three LDP SNP selection methods was small or absent, Methods 2 and 3 reduced the variation in imputation accuracy across SNPs (see Figure 2 and Additional file 5: Figure S2). As shown in Table 2 for ECA1 (and Additional file 6: Table S3, for all chromosomes), Methods 2 and 3 resulted in an increase in both the mean MAF of the selected LDP SNPs and the standard deviation of the distance between the LDP SNPs.

**Table 1:** The mean proportion of correctly imputed genotypes, as calculated in the within-population analysis of the UK dataset

**Number of SNPs**^**1**^	**bpEQ**	**bpMAF**	**lduMAF**
**Per individual**			
384	0.66 (0.52,0.93)	0.67 (0.55,0.94)	0.69 (0.55,0.92)
768	0.76 (0.59,0.94)	0.77 (0.62,0.95)	0.78 (0.59,0.96)
1K	0.79 (0.61,0.94)	0.84 (0.66,0.97)	0.83 (0.64,0.98)
2K	0.90 (0.70,0.99)	0.91 (0.71,0.99)	0.89 (0.68,0.99)
3K	0.94 (0.70,0.99)	0.95 (0.73,1.00)	0.92 (0.67,0.99)
6K	0.97 (0.79,1.00)	0.98 (0.78,1.00)	0.95 (0.75,1.00)
**Per SNP**			
384	0.66 (0.30,1.00)	0.67 (0.30,1.00)	0.69 (0.36,1.00)
768	0.76 (0.37,1.00)	0.77 (0.44,1.00)	0.78 (0.50,1.00)
1K	0.79 (0.44,1.00)	0.84 (0.50,1.00)	0.83 (0.53,1.00)
2K	0.90 (0.56,1.00)	0.91 (0.63,1.00)	0.89 (0.53,1.00)
3K	0.94 (0.68,1.00)	0.95 (0.72,1.00)	0.92 (0.66,1.00)
6K	0.97 (0.79,1.00)	0.98 (0.83,1.00)	0.95 (0.72,1.00)

Mean proportion of correctly imputed genotypes per individual or per SNP for ECA1, with minimum and maximum values in brackets (tables for all chromosomes are in Additional file 4: Table S2); ^1^total number of SNPs that would be on a genome-wide LDP of equivalent density.

**Table 2:** Properties of low density panel SNPs, as calculated in the within-population analysis of the UK dataset

**Number of SNPs**^**1**^	**Method**	**Mean (SD) MAF**	**Mean (SD) distance between SNPs (Mb)**
384	bpEQ	0.22 (0.13)	6.40 (0.09)
	bpMAF	0.25 (0.10)	6.40 (0.66)
	lduMAF	0.44 (0.06)	6.40 (2.41)
768	bpEQ	0.25 (0.15)	3.14 (0.09)
	bpMAF	0.31 (0.13)	3.14 (0.57)
	lduMAF	0.45 (0.04)	3.14 (1.65)
1K	bpEQ	0.22 (0.14)	2.41 (0.07)
	bpMAF	0.39 (0.08)	2.38 (0.61)
	lduMAF	0.45 (0.04)	2.38 (1.42)
2K	bpEQ	0.23 (0.14)	1.19 (0.06)
	bpMAF	0.28 (0.11)	1.19 (0.30)
	lduMAF	0.46 (0.04)	1.19 (1.19)
3K	bpEQ	0.23 (0.14)	0.79 (0.06)
	bpMAF	0.30 (0.12)	0.79 (0.23)
	lduMAF	0.46 (0.03)	0.79 (0.92)
6K	bpEQ	0.23 (0.14)	0.39 (0.07)
	bpMAF	0.29 (0.11)	0.39 (0.17)
	lduMAF	0.43 (0.05)	0.39 (0.63)

Properties of low density panel SNPs for ECA1 selected using three methods (tables for all chromosomes are in Additional file 6: Table S3); ^1^total number of SNPs that would be on a genome-wide LDP of equivalent density.

**Figure 3:**
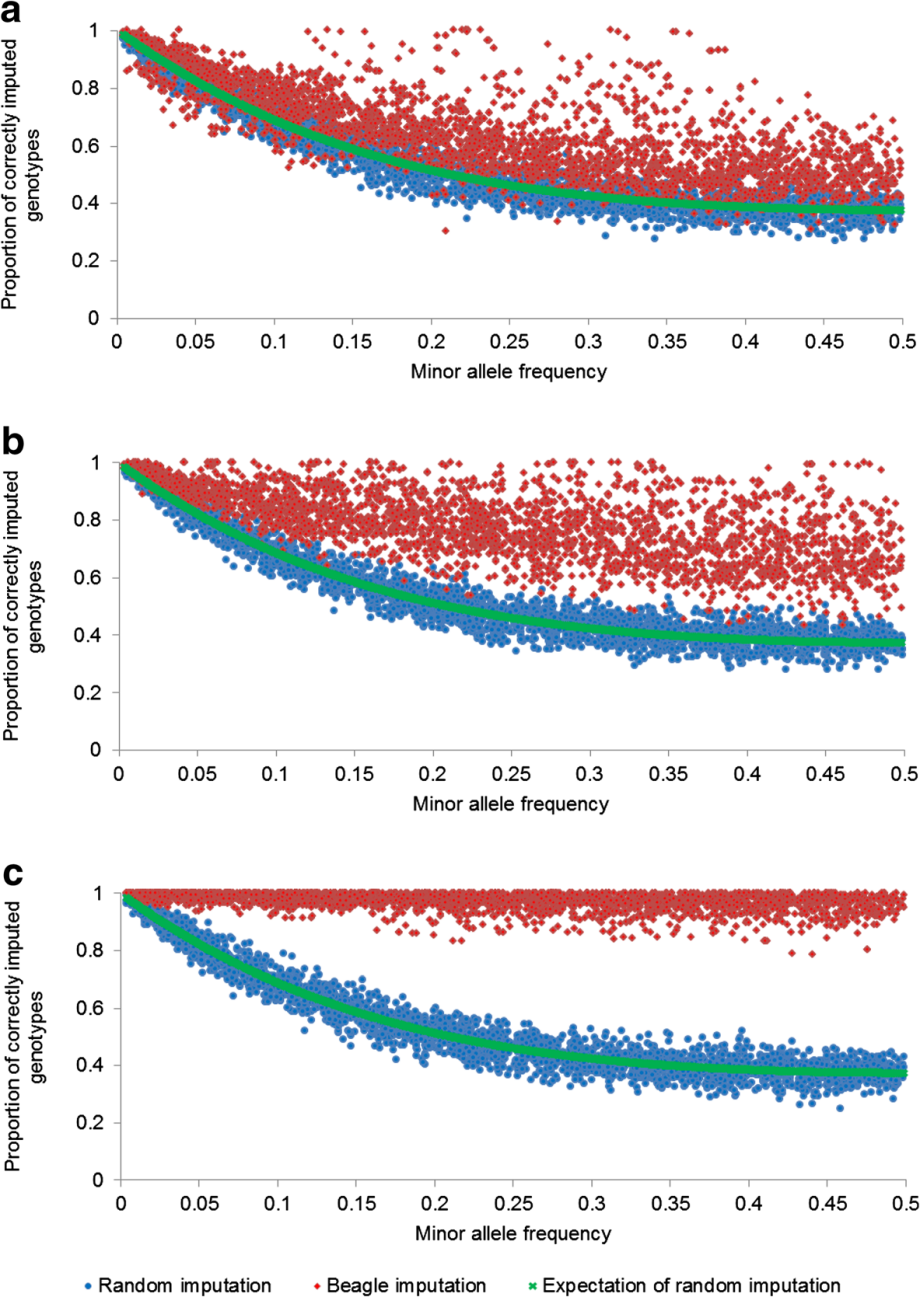
**The proportion of correctly imputed genotypes plotted against the MAF of the SNPs being imputed (calculated in the reference population) for ECA1 (bpEQ).** As calculated in the within-population analysis of the UK dataset. **a)** 384 panel; **b)** 1K panel; **c)** 6K panel.

Random imputation of genotypes at masked loci, based on allele frequencies in the reference population, quantifies the minimum imputation accuracy that can be expected. Figure 3 shows the strong dependency of the accuracy of imputation on MAF with random imputation and the results follow closely the expectation (see Methods). This relationship between MAF and imputation accuracy was less clear when BEAGLE was used for imputation, except at lower densities (Figure 3).

**Figure 4:**
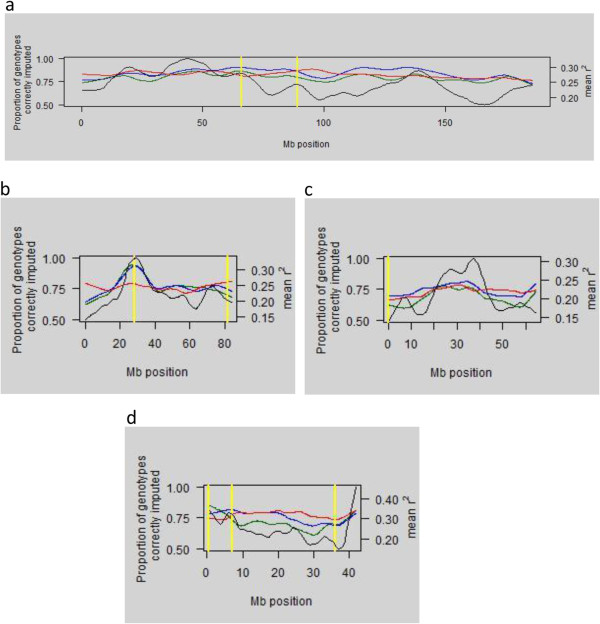
**The proportion of correctly imputed genotypes by SNP and the mean linkage disequilibrium plotted against SNP position for the 1K panel.** The figure presents Lowess curves, as calculated in R [45-48]; green = bpEQ; blue = bpMAF; red = lduMAF; black = mean linkage disequilibrium ($r_{g}^{2}$) in sliding windows of 1 Mb (with 0.5 Mb overlap); yellow = hypothesised position of the centromere. **a)** ECA1; **b)** ECA10; **c)** ECA20; **d)** ECA26.

In order to explore possible causes of differences in imputation accuracy between SNPs, imputation accuracy and average pairwise $r_{g}^{2}$ were plotted against SNP position (bp) (Figure 4). The hypothesised positions of the centromeres are also marked on the plots. Based on similarity with centromeric satellite sequences, it was assumed that the centromere position of ECA1 was located at 66 Mb or 89 Mb and of ECA10 at 28.2 Mb, although there was a second region between 81 Mb and 83 Mb that also contained some centromeric satellite-like sequences; ECA20 and ECA26 are not centromeric but regions identified for these chromosomes may represent regions that contained centromeres in the past, if the similarity with centromeric satellite sequences is real (CM Wade 2012, personal communication). Figure 4 shows considerable variation in imputation accuracy across the chromosome, which was often positively correlated with levels of LD. This variation was particularly marked for ECA10, for which a peak in imputation accuracy was observed in the region that surrounds the proposed centromere. When Method 3 (lduMAF) was used to select LDP SNPs the variation in accuracy across the chromosome was reduced, which led to a more consistent level of accuracy and a reduction in its correlation with LD levels; this corresponds to the decreased variance in imputation accuracy across SNPs observed when using this method shown in Figure 2 and Additional file 5: Figure S2. In general, the decrease in accuracy obtained with Method 3 in regions of high LD compared to Methods 1 and 2 was greater than the corresponding increase in low LD areas. This explains the inability of this method to improve mean accuracies above those achieved using Method 2 (bpMAF).

**Figure 5:**
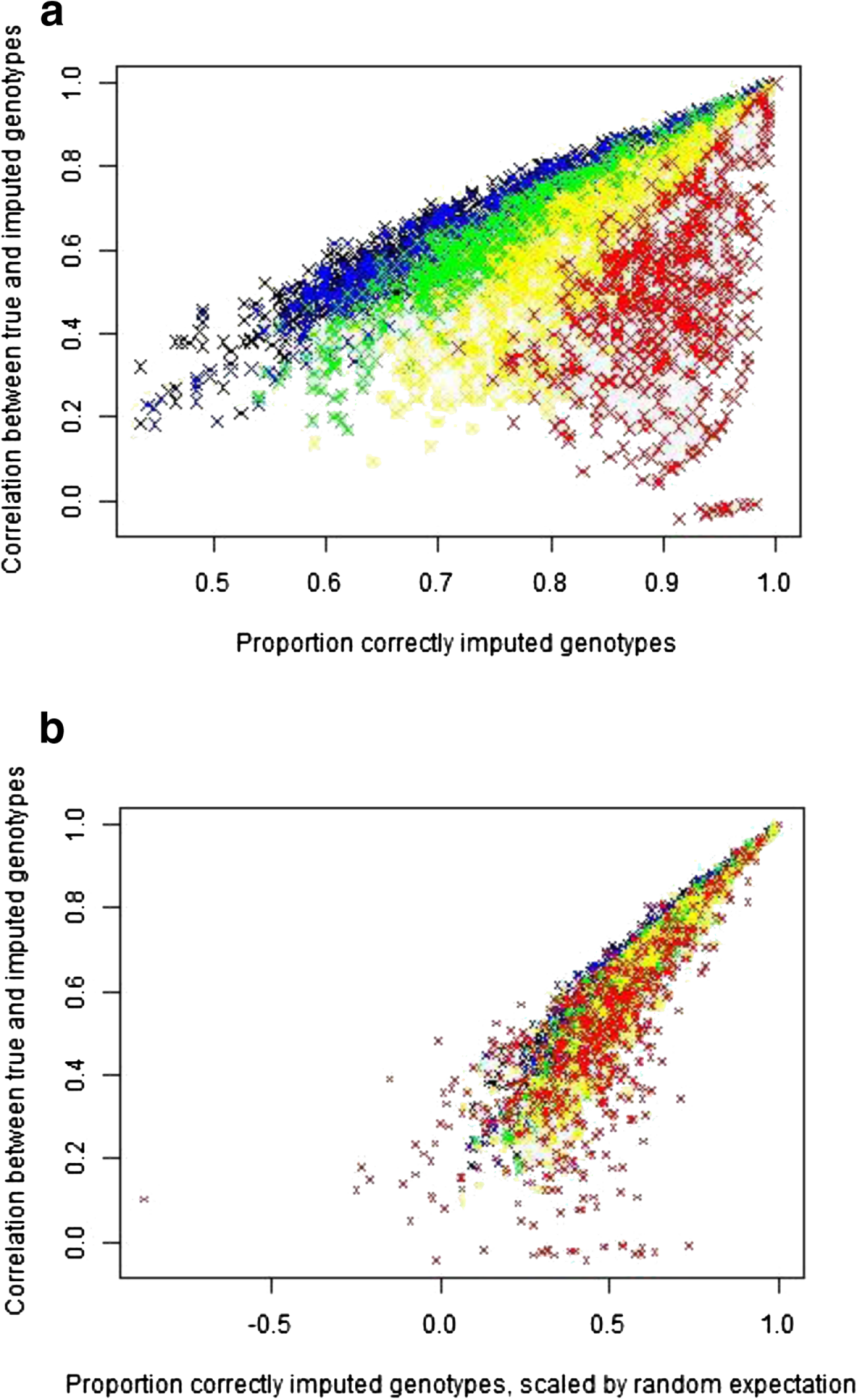
**The correlation between true and imputed genotypes by SNP. a)** Plotted against the proportion of correctly imputed genotypes; **b)** Plotted against the proportion of correctly imputed genotypes, scaled by the proportion expected from random imputation. Black = SNPs with MAF ≥ 0.40; blue = SNPs with 0.30 ≤ MAF < 0.40; green = SNPs with 0.20 ≤ MAF < 0.30; yellow = SNPs with 0.10 ≤ MAF < 0.20; red = SNPs with MAF < 0.10; data for ECA1 and 1K panel.

The correlation between true and predicted genotypes was also calculated as an alternative measure of imputation accuracy. While accuracies were generally lower when expressed as correlations, considerable differences between horses and between SNPs remained (Table 3 and Additional file 7: Table S4). A comparison of the two accuracy measures showed some correspondence but the relationship depended upon MAF (Figure 5a). Adjusting the proportion of correctly imputed genotypes for the expected proportion achievable by random imputation resulted in a much stronger relationship with the correlation between true and imputed genotypes (Figure 5b), which was almost independent of the MAF, although SNPs with a lower MAF tended to show more variation in both measures of accuracy.

**Table 3:** The mean correlation between true and predicted genotypes, as calculated in the within-population analysis of the UK dataset

**Number of SNPs**^**1**^	**Method for selection of low density SNPs**		
**per individual**	**bpEQ**	**bpMAF**	**lduMAF**
384	0.46 (0.14,0.89)	0.49 (0.20,0.91)	0.53 (0.22,0.89)
768	0.64 (0.36,0.93)	0.66 (0.38,0.93)	0.69 (0.37,0.94)
1K	0.70 (0.41,0.93)	0.78 (0.51,0.96)	0.75 (0.47,0.98)
2K	0.86 (0.53,0.99)	0.88 (0.62,0.98)	0.85 (0.52,0.99)
3K	0.92 (0.59,0.99)	0.94 (0.60,1.00)	0.88 (0.48,0.99)
6K	0.97 (0.73,1.00)	0.97 (0.71,1.00)	0.93 (0.61,1.00)
**per SNP**			
384	0.30 (-0.17,1.00)	0.32 (-0.14,1.00)	0.36 (-0.08,1.00)
768	0.52 (-0.08,1.00)	0.53 (-0.06,1.00)	0.55 (-0.05,1.00)
1K	0.60 (-0.04,1.00)	0.67 (-0.05,1.00)	0.64 (-0.05,1.00)
2K	0.81 (-0.04,1.00)	0.83 (-0.02,1.00)	0.79 (-0.02,1.00)
3K	0.89 (-0.01,1.00)	0.90 (-0.03,1.00)	0.83 (-0.02,1.00)
6K	0.95 (0.25,1.00)	0.96 (0.49,1.00)	0.90 (-0.01,1.00)

Mean correlation between true and predicted genotypes per individual or per SNP for ECA1, with minimum and maximum values in brackets (tables for all chromosomes are in Additional file 7: Table S4); ^1^total number of SNPs that would be on a genome-wide LDP of equivalent density.

### Between-population assessment of imputation accuracy

When Set B (the UK reference population) was used as the reference population for imputation in the US dataset, there was very little change in the mean proportion of correctly imputed genotypes relative to the within-population results (Table 4). With random imputation, there was no difference in accuracy between the within- and between-population analyses for ECA1 whereas a small but consistent decrease of 0.01 in the mean was seen for ECA26. This small difference is presumably due to the high correlation between the MAF of SNPs in the two populations, which was equal to 0.91 for ECA1 and 0.90 for ECA26. Imputation using BEAGLE gave a similar pattern of results, with no difference in imputation accuracy for ECA1 and a slight decrease in accuracy for ECA26 when compared to the within-population results for the UK described above.

**Table 4:** The mean proportion of correctly imputed genotypes for ECA1 and ECA26, as calculated in the between-population analysis of the US dataset with the 2K panel

**Chr**	**Imputation method**	**LDP SNP selection method**	**UK within-population assessment**^**a**^	**Between-population assessment 1**^**b**^	**Between-population assessment 2**^**c**^
ECA1	Random^d^	bpEQ	0.55	0.55	0.56
		bpMAF	0.55	0.55	0.56
		lduMAF	0.56	0.56	0.57
	Beagle^e^	bpEQ	0.90	0.90	0.92
		bpMAF	0.91	0.91	0.93
		lduMAF	0.89	0.89	0.92
ECA26	Random^f^	bpEQ	0.51	0.50	0.51
		bpMAF	0.52	0.51	0.51
		lduMAF	0.52	0.51	0.51
	Beagle^g^	bpEQ	0.82	0.81	0.86
		bpMAF	0.85	0.84	0.89
		lduMAF	0.88	0.85	0.90

^a^reference population B and test population C; ^b^reference population B and test population D + E; ^c^reference population C and test population D; ^d^SE across SNPs and samples was equal to 0.003 and 9×10^-4^ to 2×10^-3^, respectively; ^e^SE across SNPs and samples was equal to 0.001 and 2×10^-3^ to 5×10^-3^, respectively; ^f^SE across SNPs and samples was equal to 0.006 and 1×10^-3^ to 3×10^-3^, respectively; ^g^SE across SNPs and samples was equal to 2×10^-3^ to 4×10^-3^ and 4×10^-3^ to 1×10^-2^, respectively.

When Sets D and E were used as reference and test populations, respectively, imputation accuracy was slightly increased compared to that obtained for within-UK imputation, for both imputation methods (random and BEAGLE) and for all three LDP SNP sets (Table 4). This increase is probably due to the higher average relationship between horses in the US dataset compared to the UK dataset; when average genomic relationships were calculated for all samples using SNPs on ECA1 (as in [25]), the mean relationship between horses was 0.022 in the US dataset and 0.003 in the UK dataset.

## Discussion

In this study, the efficacy of imputation from low- to high-density in Thoroughbred horses was investigated and three methods for selecting the LDP SNP were compared. Two measures were used to assess imputation accuracy, the proportion of correctly imputed genotypes and the correlation between true and imputed genotypes. While these two measures were correlated, the proportion of correctly imputed genotypes was related to the MAF of the imputed SNPs. Adjusting the proportion of correctly imputed genotypes by the expected accuracy using random imputation (as in [7]) weakened the relationship with MAF, but emphasized the greater variation in imputation accuracies for SNPs with a low MAF. In contrast, the correlation measure provided an assessment of imputation accuracy that was less dependent on MAF. This property makes it preferable for comparing methods, which is a similar conclusion to that of Hickey et al. [8] in their study on maize. However, because accuracy expressed as the proportion of correctly imputed genotypes is more easily compared to results of other studies, it is also presented here. The haplotype phasing and imputation program used here (BEAGLE) has been shown to perform similarly to other available software [7,17,39] and therefore the results presented are considered to be representative.

### Factors affecting imputation accuracy

Increasing the SNP density of the LDP serves to reduce the considerable range in imputation accuracy between SNPs and between horses. For example, at the lowest density, some SNPs were imputed correctly for all horses, whereas other SNPs were correct in as few as 24% of horses (ECA10 bpEQ results). Increasing the density of the LDP led to an increase in the minimum accuracy across SNPs, so for ECA10 the 6K LDP gave a minimum accuracy of 83%. A similar effect was observed across horses. By calculating marker densities normalised by N_e_, results can be compared to those from other studies and species. Using a 2K low-density panel in Border Leicester sheep, equivalent to a SNP density of 0.23N_e_/Morgan (assuming N_e_ = 242 [40] and a total genome length of 36.3 Morgans [41]), Hayes et al. [7] achieved an imputation accuracy of approximately 0.73 (measured by the unadjusted proportion of correctly imputed genotypes). Thus, our results using the 0.24N_e_/Morgan bpMAF SNP panel (the 1K panel) compare favourably, with accuracies that ranged from 0.74 to 0.84 across chromosomes. A study on Jersey cattle that used equivalent SNP densities also obtained accuracies in the range of 0.7 to 0.8 [18].

Random imputation resulted in a direct and predictable relationship between the MAF of SNPs and the accuracy with which they were imputed. This relationship was less evident when using BEAGLE for imputation, except at the lowest LDP SNP densities, for which the amount of information available from LD was presumably low. Using a genetic algorithm to preferentially select LDP SNPs that are more informative, i.e. having a high MAF, while simultaneously ensuring consistent coverage across the chromosome (bpMAF), achieved a small but consistent increase in the proportion of correctly imputed genotypes, with increases ranging from 0.6% to 5.3% for the 1K SNP panel. Further small improvements might be obtained by differential weighting of the two parts of the objective function used, so making MAF of greater or lesser importance. Using method bpMAF to select LDP SNPs also resulted in a decrease in the variation in imputation accuracy across SNPs compared to the bpEQ method.

A major source of the variation in imputation accuracy across SNPs was the extent of LD, with variation both between and within chromosomes. There was a tendency for imputation accuracies to be higher for the longer chromosomes (ECA1 and ECA10) and this coincides with the higher average LD of these chromosomes, shown by Corbin et al. [42]. Within chromosomes, SNPs in regions of high LD were imputed more accurately than SNPs in regions of low LD. The strength of this relationship differed between the four chromosomes and was strongest for ECA10, where the region of highest LD (and imputation accuracy) coincided with the hypothesised position of the centromere. This relationship between LD and the accuracy of imputation was not observed for ECA1, which suggests that the processes underlying the observed LD may be important. When locations of SNPs were scaled based on LD map distance (as a proxy for linkage map distance) prior to their selection for the LDP, as in Method 3 (lduMAF), this relationship between LD and imputation accuracy was broken down (Figure 4). This resulted in a decrease in the variance of imputation accuracy between SNPs. However, the change in mean imputation accuracy relative to Method 2 (bpMAF) was small and inconsistent, with an increase in accuracy for ECA26 and a decrease for ECA1.

Using lduMAF to select LDP SNPs increased imputation accuracy for SNPs that were in low LD regions due to a greater concentration of SNPs selected in these regions, but the decrease in accuracy for SNPs in high LD regions was relatively greater, resulting in a trend for the mean accuracy to be reduced. The relatively poor performance of the lduMAF may be due in part to the use of *D′* rather than *r*^*2*^ in the Malecot model. Constructing an LDP including consideration of *r*^*2*^ may give better results, although *r*^*2*^ is already low in Thoroughbreds at the densities used for the LDP [42]. An additional benefit may also be obtained if the lduMAF approach was applied at a genome-wide level, such that the number of LDP SNPs per chromosome was proportional to its LD map distance, rather than its base pair length as in the current implementation. If pedigree were available and if genotyping were to become common in the Thoroughbred, constructing accurate maps of intra-chromosomal linkage in Morgans derived from the phasing carried out within the imputation process would be straightforward (JM Hickey 2012, personal communication). Such maps could then be used directly to produce an LDP better able to capture recombination events and hence improve imputation accuracy.

The results of analyses presented here suggest that there is some ambiguity over which properties of imputation are most important when assessing efficacy. Does the utility for imputation argue for choosing LDP SNPs to maximise the mean imputation accuracy, or to maximise a minimum (or low percentile of) imputation accuracy; as judged by SNPs or by horses? Concern over lower percentiles will place more value on reducing the variance of imputation errors. While the answer lies in the intended use of the imputed genotypes, it would be useful to have some generic assessment of imputation performance. One solution might be to use a utility function such as the area under the curve obtained from plotting SNP correlation against SNP position, as in Figure 4. The development of a whole-genome measure of imputation success, integrating location and accuracy, would allow for a more comprehensive and quantitative comparison of the different LDP SNP sets used in this study, in particular the relative usefulness of the novel lduMAF approach.

### Between-population assessment of imputation accuracy

Transferability across breeds and across countries within breeds is an important consideration when designing a LDP. Here, data from a cohort of US Thoroughbreds was used to evaluate the impact of geographical origin on the efficacy of imputation. While comparisons of within-UK, within-US and UK to US imputations involved reference and test populations of different sizes, studies have shown that the size of the reference population does not have a major impact on imputation accuracy for the sizes used here [7,10]. In this study, replacing the UK test population with a sample of horses from a different geographical area (the US) had a negligible impact on imputation accuracy. This implies that similar LD patterns exist in both populations, which in turn indicates that the genetic differentiation between the UK and US populations is small, or that a similar LD structure exists due to a common recombination background, or both. The high correlation between MAF of SNPs in the two populations lends some credence to the former argument, while the relationship between LD and the centromere position in ECA10 suggests that the latter is also relevant. One can assume that the US and UK populations share some similarity, given the relatively recent breed formation (around 30 generations ago) combined with cross-border matings and the relatively small number of founders (effective number of studbook founders of 28.2 [43]). When average genomic relationships were calculated for all samples using SNPs on ECA1 (as in [25]), the mean relationship of horses from the US with those from the UK was -0.01.

Replacing both the UK reference and test populations with horses from the US resulted in an increase in imputation accuracy for the US test population compared to using UK horses as the reference population. This was despite the fact that the LDP SNPs were selected using a UK population sample. One explanation for this increase in accuracy is the higher average relationships in the US dataset, which has been shown to improve imputation accuracy [8]. However, the fact that there was no difference in the relative increase in imputation accuracy between the bpEQ LDPs (which is not population dependent) and the bpMAF and lduMAF LDPs (which do depend upon the UK dataset), suggests that the LDP SNP sets are equally appropriate for both populations. Whether this result also holds across breeds is more doubtful. The frequent sharing of major haplotypes between diverse horse breeds [27] suggests that a certain degree of accuracy should be maintained, but further indications of the likely efficacy of imputation across breeds may be sought by comparing allele frequencies of the breeds in question.

### Determining optimal LDP size

The value of imputed genotypes depends on both their accuracy and their purpose. Daetwyler et al. [44] observed that the accuracy of genomic estimated breeding values (GEBV) achieved with SNP genotypes imputed from a sparse set of markers, as a percentage of that achieved for the dense SNP genotypes, was in all cases greater than the proportion of correctly imputed genotypes. Specifically, when 87.8% of missing genotypes were correctly imputed, the accuracy of GEBV was reduced by only 5%. Furthermore, the imputation accuracy greater than 0.90 that was achieved here for the 3K panel is very similar to that reported by Weigel et al. [10] in a study on daughter pregnancy rate, which resulted in a GEBV accuracy of 0.642 when imputation was used (from a LDP of 2942 SNPs), compared to 0.674 when all SNPs were genotyped (42 552 SNPs). Given these results, it is likely that an LDP of 2K to 3K SNPs could lead to sufficiently high imputation accuracies to be useful in Thoroughbred horses.

Any loss of accuracy in imputation that occurs as a result of using lower density SNP panels must also be considered alongside the cost savings that would be achieved. Part of the accuracy lost might be recovered if the pedigrees of genotyped individuals were available [14]. However, based on estimated genotyping costs for 384 to 2K SNPs, with 1K and 2K SNP panel prices based on a custom chip construction, and for 3072 to 6K SNPs, based on the iSelect Infinium Assay, there is no difference in cost between genotyping 768 and 2K SNPs, or between genotyping 3072 and 6K SNPs (Source: GeneSeek representative, 2012). Therefore, the logical choice is between a 384, a 2K and a 6K SNP panel, with these options offering 42, 84 and 96% of the accuracy in imputation for 17%, 29% and 40% of the cost of the equine 70K SNP chip (used because the Equine SNP50 BeadChip is no longer available to purchase), respectively. Whilst the cost increases by the same amount (US$20) from 384 to 2K and then from 2K to 6K, the increase in imputation accuracy is more than three times greater from 384 to 2K than from 2K to 6K, suggesting that a 2K SNP panel represents better value for money. However, specific uses may demand specific accuracies, in which case cost could be less important.

## Conclusions

The results of this study show that it is possible to impute genotypes from low- to high- density in Thoroughbred horses with reasonable to high accuracy. An investigation of the source of differences in imputation accuracy revealed dependence on the MAF of the SNPs being imputed, and on the underlying LD structure. While equidistant LDP SNPs worked well, optimising LDP SNP selection to increase their MAF was advantageous leading to increased imputation accuracy, even when LDPs were subsequently used in a population of different geographical origin. By using LD map distance instead of physical distance to select LDP SNPs, differences in imputation accuracy between SNPs were reduced. Whereas a 1K SNP panel was generally sufficient to ensure that more than 80% of genotypes were correctly imputed, inference from other studies suggests that a 2 to 3K SNP panel would ensure that the subsequent loss in accuracy for, for example, genomic prediction was minimal [10,44]. Furthermore, the relationship between accuracy and genotyping costs for the different LDPs, suggest that a 2K SNP panel would represent good value for money for Thoroughbreds. More work is needed to evaluate the impact of between-breed differences on imputation accuracy. Imputation makes it possible to use low-density SNP panels as a low cost alternative to high-density genotyping but it also provides a means to combine datasets from different genotyping platforms, a possibility that will become necessary as researchers are starting to use the recently developed equine 70K SNP chip.

## Competing interests

The authors declare that they have no competing interests.

## Authors’ contributions

LJC conceived the study, carried out the analyses (with the exception of the writing and running of the genetic algorithm used to select LDP SNPs in Methods 2 and 3, which was done by AK) and wrote the manuscript; JAW, SCB (The Roslin Institute) and AK provided advice on the study design and analyses, and contributed to the manuscript; SCB (Animal Health Trust), JES and MV coordinated the collection of all the samples, provided the genotype data, and collated information on genotyping costs. All authors read and approved the final manuscript.

## Supplementary Material

Additional file 1: Table S1 Quality control criteria implemented on genotype data and number of SNPs discarded at each step. This table contains details of SNP exclusions made on the basis of quality control carried out using the Illumina GenomeStudio genotyping module. Click here for file

Additional file 2 **Calculation of LD maps.** This document contains further details of the method used to calculate SNP positions in LDU for LDP SNP selection Method 3, including settings used to run the LDMAP program. Click here for file

Additional file 3: Figure S1 LD maps. This document contains figures showing the relationship between physical map distance (Mb) and map distance in LDU for chromosomes 1, 10, 20 and 26. Click here for file

Additional file 4: Table S2 The mean proportion of correctly imputed genotypes, as calculated in the within-population analysis of the UK dataset. The data provided represent the results of imputation from LDPs with SNPs selected by Methods 1 to 3, expressed as the mean proportion of correctly imputed genotypes, both per individual and per SNP. Results are shown for chromosomes 1, 10, 20 and 26. Click here for file

Additional file 5: Figure S2 The mean proportion of correctly imputed genotypes and its variance across SNPs, as calculated in the within-population analysis of the UK dataset. The figures provided show the results of imputation from LDPs with SNPs selected by Methods 1 to 3, expressed as the mean proportion of correctly imputed genotypes per SNP and plotted against the total number of SNPs on a genome-wide LDP of equivalent density. Results are shown for chromosomes 1, 10, 20 and 26. Click here for file

Additional file 6: Table S3 Properties of low density panel SNPs, as calculated in the within-population analysis of the UK dataset. The data represent properties of LDP SNPs as selected by Methods 1 to 3. Results are shown for chromosomes 1, 10, 20 and 26. Click here for file

Additional file 7: Table S4 The mean correlation between true and predicted genotypes, as calculated in the within-population analysis of the UK dataset. The data provided represent the results of imputation from LDPs with SNPs selected by Methods 1 to 3, expressed as the mean correlation between true and predicted genotypes, both per individual and per SNP. Results are shown for chromosomes 1, 10, 20 and 26. Click here for file
