# Supplementary material for: The utility of low-density genotyping for imputation in the Thoroughbred horse
Source: Genet Sel Evol. 2014 Feb 4;46(1):9. doi: 10.1186/1297-9686-46-9 (PMC3930001; doi:10.1186/1297-9686-46-9)
Supplement: Additional file 1: Table S1 — Quality control criteria implemented on genotype data and number of SNPs discarded at each step. This table contains details of SNP exclusions made on the basis of quality control carried out using the Illumina GenomeStudio genotyping module. [file 1297-9686-46-9-S1.pdf]

Table S1 Quality control criteria implemented on genotype data and the number of SNPs discarded at each step. This quality control was carried out as part of a larger study involving a total of 1342 samples.

**Metric examined**	**Purpose of metric applied**	**Threshold applied**	**Number of SNP removed at this stage**
Cluster separation[I]	Removal of SNPs with inadequately defined clusters	<0.25 discarded	1319
Call frequency	Removal of SNPs which were not called in a minimum number of samples	<98% call rate discarded	2380
AB R Mean[II]	Removal of SNPs with low intensity data	<0.15 discarded	7
AB T Mean[III]	Removal of SNPs for which the heterozygote cluster was not well separated from the homozygote clusters	<0.2 and >0.8 discarded	20
Heterozygote excess	Removal of SNPs which deviate significantly from Hardy-Weinberg equilibrium	Heterozygosity of <-0.3 and >0.1 discarded	149
ECAX markers	Removal of X chromosome markers for which the males were called heterozygous	Each marker assessed individually	20

[I]Measures the separation between the three genotype clusters and varies from 0 to 1

[II]Mean normalized intensity of the heterozygote cluster, with values increasing from 0

[III]Mean of the normalized theta values of the heterozygote cluster, with values ranging from 0-1. Values of <0.2 and >0.8 indicate that the heterozygote cluster has shifted towards the homozygotes.
