# Supplementary material for: The utility of low-density genotyping for imputation in the Thoroughbred horse
Source: Genet Sel Evol. 2014 Feb 4;46(1):9. doi: 10.1186/1297-9686-46-9 (PMC3930001; doi:10.1186/1297-9686-46-9)
Supplement: Additional file 2 — Calculation of LD maps. This document contains further details of the method used to calculate SNP positions in LDU for LDP SNP selection Method 3, including settings used to run the LDMAP program. [file 1297-9686-46-9-S2.pdf]

Additional file 2 Calculation of LD Maps

The LDMAP program described and developed by Maniatis et al. (2002) [1] was used to construct an LD map for each chromosome using the diplotypes of samples in Set A. First, LDMAP computes the absolute D′-statistic [2] between all pairs of markers. For map construction, each of the chromosomes was divided into several overlapping segments with a maximum of 250 SNPs per segment and an overlap of 50 SNPs. The number of segments per chromosome ranged from four (ECA26) to 15 (ECA1). For each segment, LDMAP fits the Malecot model to D′ vs. inter-marker distance (d) data in kilobases. This quantifies the average rate of decline of LD for the segment which is a useful starting value to compute the interval-specific estimates used in LD map construction.

Using the syntax and descriptions of Maniatis et al (2002) [1], the Malecot prediction of association is: $\rho = (1 - L)Me^{-\varepsilon d} + L$, where L is the bias at large distances, M is the proportion of the youngest haplotype that is monophyletic and ε is the exponential decline of ρ with physical distance d [1]. For LD map construction, the LDMAP program estimates the ε and M parameters in the model for each interval (between adjacent SNPs) in the map, using data from marker pairs that include the interval in the sliding windows. Here, three rounds of iteration were performed. In the first round, the ε parameter was estimated with M set equal to 0.5. In the second round, both ε and M were estimated, with the starting values taken from the first round. The third round ensured convergence. The third Malecot model parameter L was kept constant at a value predicted as reported by Morton et al. (2001) [3] and generated by the program internally. Any larger interval that includes the interval being estimated contains some information about ε unless the markers in the pair are at such a large distance that they contain no more useful information about LD. Here, the maximum distance between any pair was set to 10Mb.

Again, using the syntax and descriptions of Maniatis et al (2002) [1], the length of the ith interval is $\varepsilon_i d_i$ LDU, where ε_i estimates the Malecot parameter and d_i is the length of the interval on the physical map in kilobases; a chromosome has a total of $\sum_i \varepsilon_i d_i$ LDU [1]. Once all SNPs in all segments have been mapped, the fit of the completed LD map is checked in a final round of iterations and parameter estimates. In cases in which SNPs were allocated the same position in the LD map, a small addition was made to subsequent locus positions (10^{-6}) before entering the SNP locations in the LDP SNP selection algorithm in order that

SNP order (according to the physical map) was maintained. Linkage disequilibrium maps are shown in Additional file 3, Figure S1.

1. Maniatis N, Collins A, Xu C-F, McCarthy LC, Hewett DR, Tapper W, Ennis S, Ke X, Morton NE: **The first linkage disequilibrium (LD) maps: Delineation of hot and cold blocks by diplotype analysis.** *Proc Nat Acad Sci* 2002, **99:**2228-2233.
2. Lewontin RC: **Interaction of selection and linkage. I. General considerations - Heterotic models.** *Genetics* 1964, **49:**49-67.
3. Morton NE, Zhang W, Taillon-Miller P, Ennis S, Kwok P-Y, Collins A: **The optimal measure of allelic association.** *Proc Nat Acad Sci* 2001, **98:**5217-5221.
