# Supplementary figures and images for: The utility of low-density genotyping for imputation in the Thoroughbred horse
Source: Genet Sel Evol. 2014 Feb 4;46(1):9. doi: 10.1186/1297-9686-46-9 (PMC3930001; doi:10.1186/1297-9686-46-9)

**Figure S1 LD Maps**

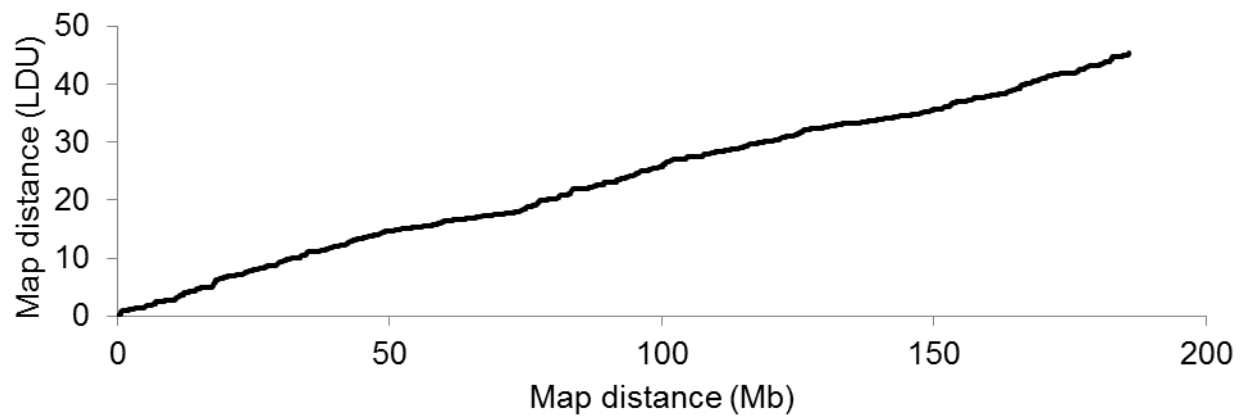

a) ECA1

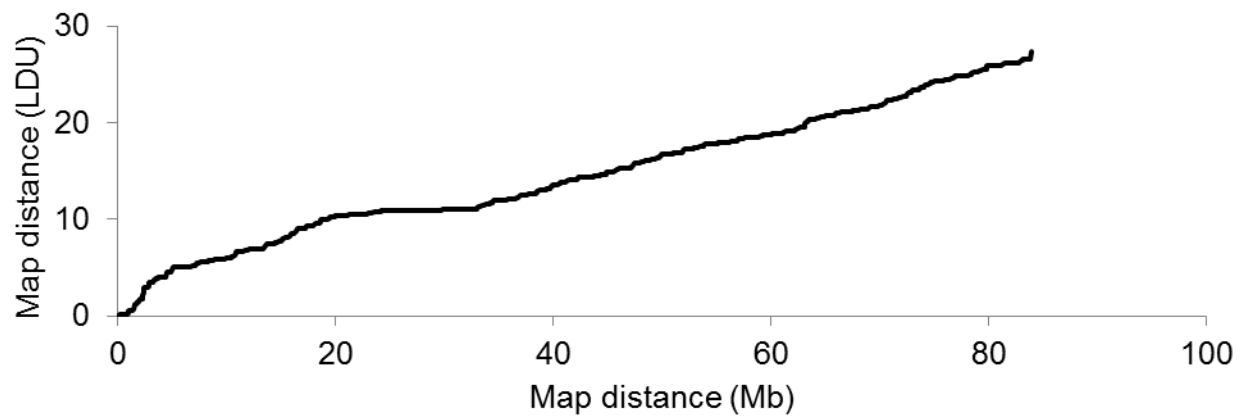

b) ECA10

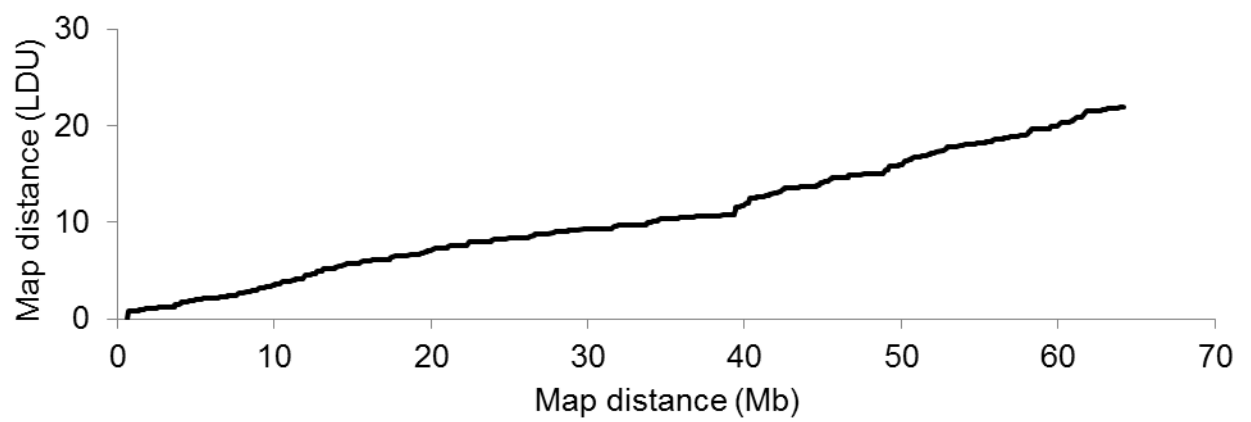

c) ECA20

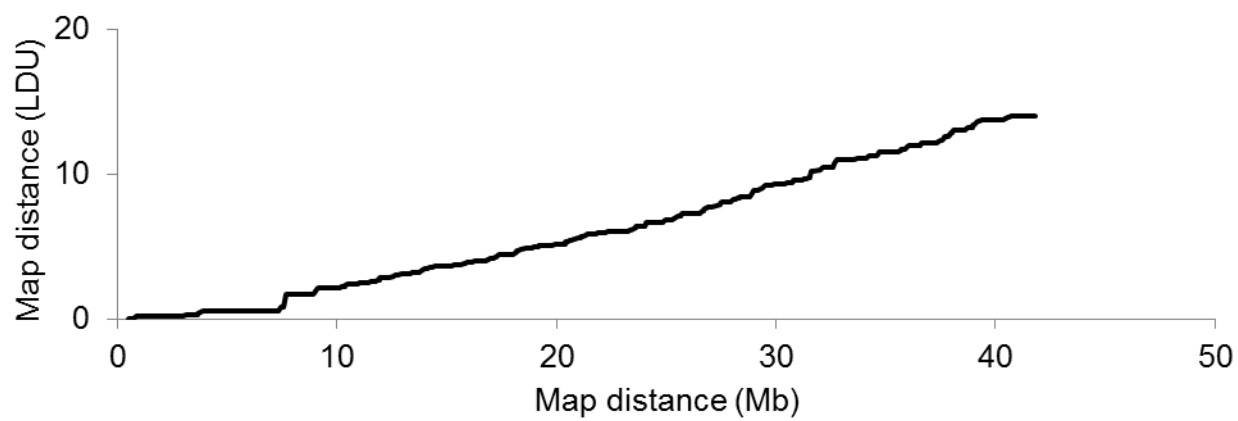

d) ECA26

Supplement: Additional file 3: Figure S1 — LD maps. This document contains figures showing the relationship between physical map distance (Mb) and map distance in LDU for chromosomes 1, 10, 20 and 26. [file 1297-9686-46-9-S3.pdf]
