# Supplementary material for: The utility of low-density genotyping for imputation in the Thoroughbred horse
Source: Genet Sel Evol. 2014 Feb 4;46(1):9. doi: 10.1186/1297-9686-46-9 (PMC3930001; doi:10.1186/1297-9686-46-9)
Supplement: Additional file 4: Table S2 — The mean proportion of correctly imputed genotypes, as calculated in the within-population analysis of the UK dataset. The data provided represent the results of imputation from LDPs with SNPs selected by Methods 1 to 3, expressed as the mean proportion of correctly imputed genotypes, both per individual and per SNP. Results are shown for chromosomes 1, 10, 20 and 26. [file 1297-9686-46-9-S4.pdf]

Table S2 The mean proportion of correctly imputed genotypes, as calculated in the within-population analysis of the UK dataset

a) Per individual

Chromosome	Number of SNPs[1]	bpEQ	bpMAF	lduMAF
**ECA1**	384	0.66 (0.52,0.93)	0.67 (0.55,0.94)	0.69 (0.55,0.92)
	768	0.76 (0.59,0.94)	0.77 (0.62,0.95)	0.78 (0.59,0.96)
	1K	0.79 (0.61,0.94)	0.84 (0.66,0.97)	0.83 (0.64,0.98)
	2K	0.90 (0.70,0.99)	0.91 (0.71,0.99)	0.89 (0.68,0.99)
	3K	0.94 (0.70,0.99)	0.95 (0.73,1.00)	0.92 (0.67,0.99)
	6K	0.97 (0.79,1.00)	0.98 (0.78,1.00)	0.95 (0.75,1.00)
**ECA10**	384	0.65 (0.51,0.99)	0.63 (0.41,0.99)	0.62 (0.49,0.96)
	768	0.71 (0.53,0.98)	0.70 (0.52,0.99)	0.71 (0.51,0.99)
	1K	0.77 (0.61,0.98)	0.78 (0.57,0.98)	0.76 (0.47,0.99)
	2K	0.87 (0.70,0.99)	0.88 (0.70,0.99)	0.90 (0.64,1.00)
	3K	0.92 (0.78,1.00)	0.93 (0.83,1.00)	0.93 (0.76,1.00)
	6K	0.97 (0.86,1.00)	0.97 (0.91,1.00)	0.96 (0.85,1.00)
**ECA20**	384	0.59 (0.45,0.90)	0.59 (0.45,0.87)	0.60 (0.43,0.87)
	768	0.67 (0.51,0.93)	0.68 (0.51,0.93)	0.68 (0.50,0.94)
	1K	0.69 (0.48,0.94)	0.74 (0.55,0.99)	0.74 (0.53,0.98)
	2K	0.82 (0.61,0.98)	0.85 (0.66,0.99)	0.87 (0.68,0.99)
	3K	0.90 (0.71,0.99)	0.92 (0.76,0.99)	0.92 (0.67,1.00)
	6K	0.96 (0.86,1.00)	0.96 (0.90,1.00)	0.95 (0.85,1.00)
**ECA26**	384	0.59 (0.38,0.93)	0.60 (0.41,0.93)	0.62 (0.40,0.98)
	768	0.70 (0.51,0.98)	0.72 (0.50,0.95)	0.72 (0.49,0.98)
	1K	0.71 (0.50,0.96)	0.76 (0.53,0.98)	0.78 (0.55,1.00)
	2K	0.82 (0.56,1.00)	0.85 (0.56,1.00)	0.88 (0.54,1.00)
	3K	0.92 (0.62,1.00)	0.92 (0.72,1.00)	0.92 (0.65,1.00)
	6K	0.96 (0.80,1.00)	0.97 (0.81,1.00)	0.94 (0.61,1.00)

Mean proportion of correctly imputed genotypes per individual, with minimum and maximum values in brackets; [1]total number of SNPs that would be on a genome-wide LDP of equivalent density.

b) Per SNP

Chromosome	Number of SNPs[1]	bpEQ	bpMAF	lduMAF
**ECA1**	384	0.66 (0.30,1.00)	0.67 (0.30,1.00)	0.69 (0.36,1.00)
	768	0.76 (0.37,1.00)	0.77 (0.44,1.00)	0.78 (0.50,1.00)
	1K	0.79 (0.44,1.00)	0.84 (0.50,1.00)	0.83 (0.53,1.00)
	2K	0.90 (0.56,1.00)	0.91 (0.63,1.00)	0.89 (0.53,1.00)
	3K	0.94 (0.68,1.00)	0.95 (0.72,1.00)	0.92 (0.66,1.00)
	6K	0.97 (0.79,1.00)	0.98 (0.83,1.00)	0.95 (0.72,1.00)
**ECA10**	384	0.65 (0.24,1.00)	0.63 (0.31,1.00)	0.62 (0.34,1.00)
	768	0.71 (0.32,1.00)	0.70 (0.35,1.00)	0.71 (0.40,1.00)
	1K	0.77 (0.36,1.00)	0.78 (0.36,1.00)	0.76 (0.44,1.00)
	2K	0.87 (0.44,1.00)	0.88 (0.47,1.00)	0.90 (0.66,1.00)
	3K	0.92 (0.48,1.00)	0.93 (0.45,1.00)	0.93 (0.74,1.00)
	6K	0.97 (0.64,1.00)	0.97 (0.65,1.00)	0.96 (0.74,1.00)
**ECA20**	384	0.59 (0.33,1.00)	0.59 (0.34,1.00)	0.60 (0.34,1.00)
	768	0.67 (0.37,1.00)	0.68 (0.35,1.00)	0.68 (0.40,1.00)
	1K	0.69 (0.39,1.00)	0.74 (0.48,1.00)	0.74 (0.44,1.00)
	2K	0.82 (0.57,1.00)	0.85 (0.56,1.00)	0.87 (0.60,1.00)
	3K	0.90 (0.66,1.00)	0.92 (0.75,1.00)	0.92 (0.69,1.00)
	6K	0.96 (0.83,1.00)	0.96 (0.86,1.00)	0.95 (0.82,1.00)
**ECA26**	384	0.59 (0.27,1.00)	0.60 (0.31,1.00)	0.62 (0.34,1.00)
	768	0.70 (0.39,1.00)	0.72 (0.42,1.00)	0.72 (0.42,1.00)
	1K	0.71 (0.36,1.00)	0.76 (0.47,1.00)	0.78 (0.52,1.00)
	2K	0.82 (0.48,1.00)	0.85 (0.52,1.00)	0.88 (0.61,1.00)
	3K	0.92 (0.71,1.00)	0.92 (0.71,1.00)	0.92 (0.77,1.00)
	6K	0.96 (0.82,1.00)	0.97 (0.87,1.00)	0.94 (0.84,1.00)

Mean proportion of correctly imputed genotypes per SNP, with minimum and maximum values in brackets; [1]total number of SNPs that would be on a genome-wide LDP of equivalent density.
