# Supplementary material for: The utility of low-density genotyping for imputation in the Thoroughbred horse
Source: Genet Sel Evol. 2014 Feb 4;46(1):9. doi: 10.1186/1297-9686-46-9 (PMC3930001; doi:10.1186/1297-9686-46-9)
Supplement: Additional file 5: Figure S2 — The mean proportion of correctly imputed genotypes and its variance across SNPs, as calculated in the within-population analysis of the UK dataset. The figures provided show the results of imputation from LDPs with SNPs selected by Methods 1 to 3, expressed as the mean proportion of correctly imputed genotypes per SNP and plotted against the total number of SNPs on a genome-wide LDP of equivalent density. Results are shown for chromosomes 1, 10, 20 and 26. [file 1297-9686-46-9-S5.pdf]

Figure S2 The mean proportion of correctly imputed genotypes and its variance across SNPs

As calculated in the within-population analysis of the UK dataset and plotted against the total number of SNPs that would be on a genome-wide LDP of equivalent density.

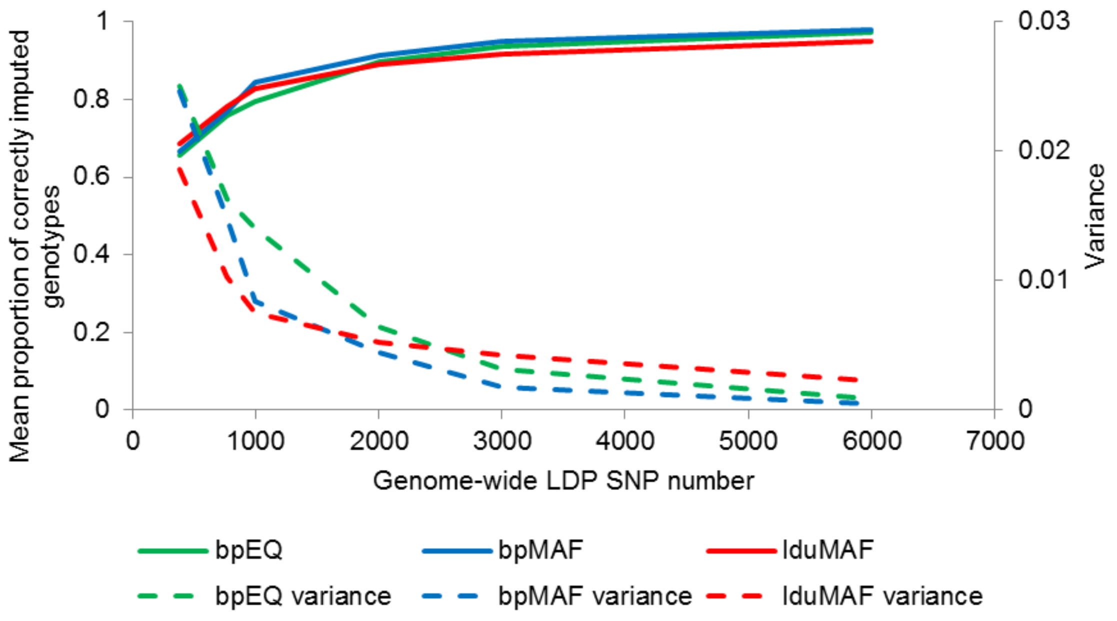

a) ECA1

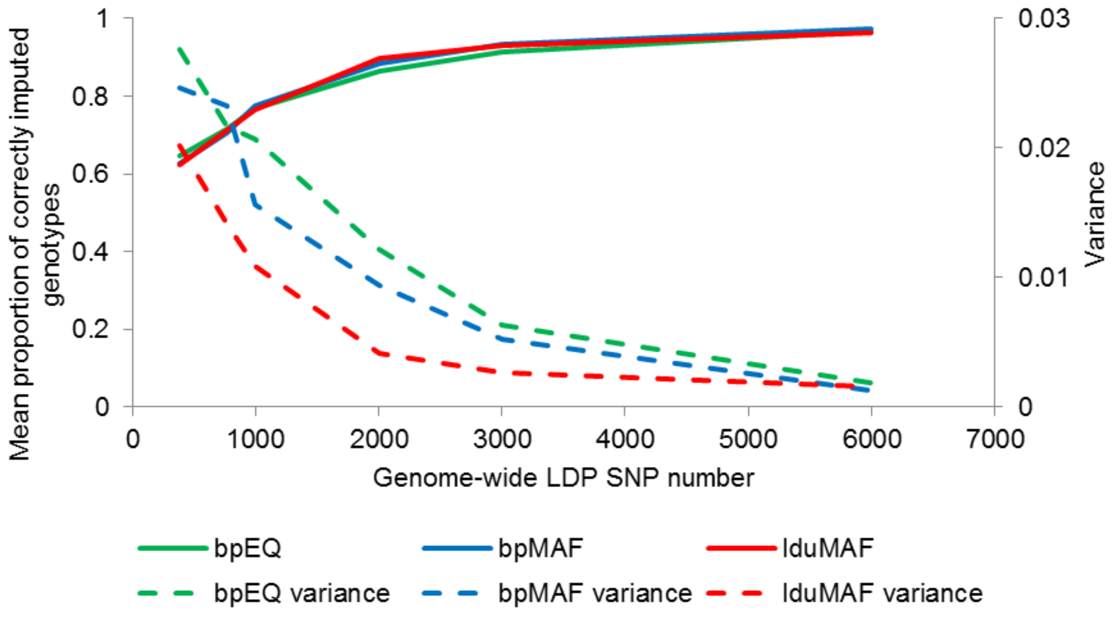

b) ECA10

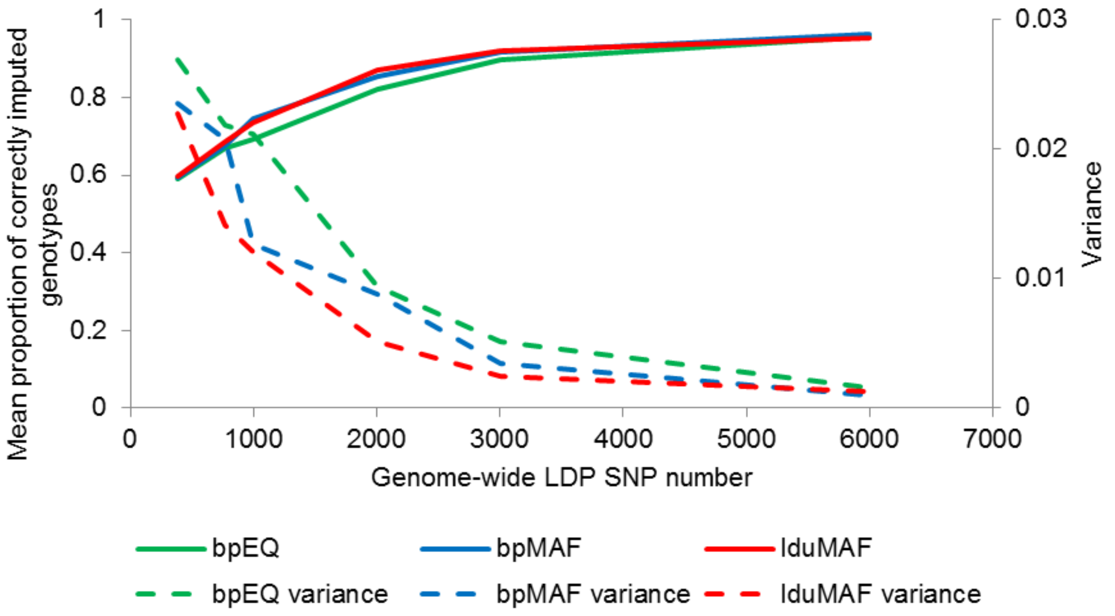

c) ECA20

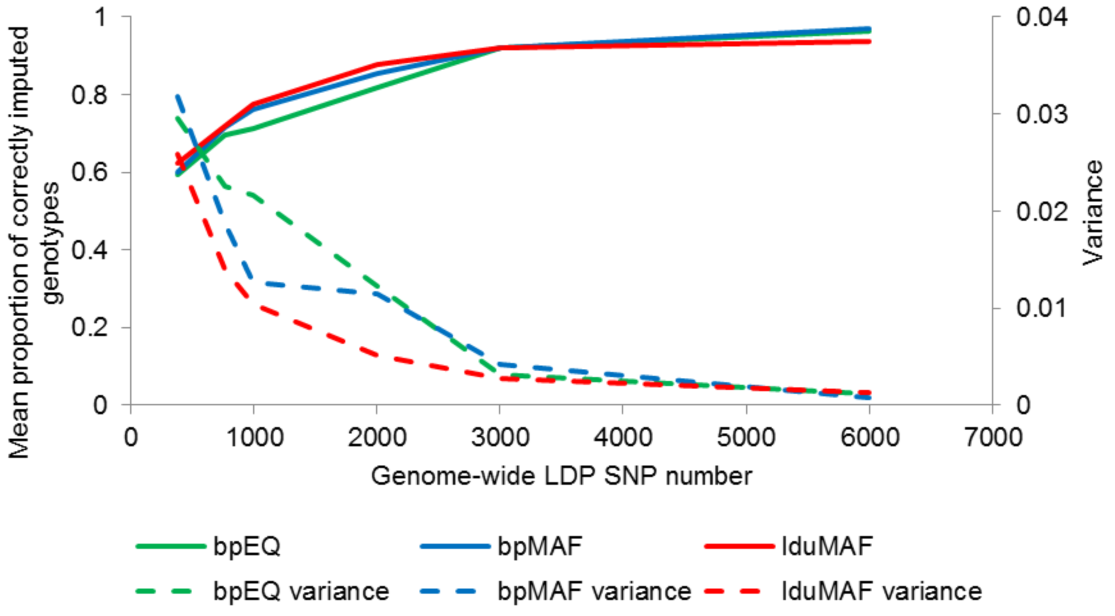

d) ECA26
