# Supplementary material for: The utility of low-density genotyping for imputation in the Thoroughbred horse
Source: Genet Sel Evol. 2014 Feb 4;46(1):9. doi: 10.1186/1297-9686-46-9 (PMC3930001; doi:10.1186/1297-9686-46-9)
Supplement: Additional file 6: Table S3 — Properties of low density panel SNPs, as calculated in the within-population analysis of the UK dataset. The data represent properties of LDP SNPs as selected by Methods 1 to 3. Results are shown for chromosomes 1, 10, 20 and 26. [file 1297-9686-46-9-S6.pdf]

Table S3 Properties of low density panel SNPs, as calculated in the within-population analysis of the UK dataset

a) ECA1

**Number of SNPs**[I]	**Method**	**Mean (SD) MAF**	**Mean (SD) distance between SNPs (Mb)**
384	bpEQ	0.22 (0.13)	6.40 (0.09)
	bpMAF	0.25 (0.10)	6.40 (0.66)
	lduMAF	0.44 (0.06)	6.40 (2.41)
768	bpEQ	0.25 (0.15)	3.14 (0.09)
	bpMAF	0.31 (0.13)	3.14 (0.57)
	lduMAF	0.45 (0.04)	3.14 (1.65)
1K	bpEQ	0.22 (0.14)	2.41 (0.07)
	bpMAF	0.39 (0.08)	2.38 (0.61)
	lduMAF	0.45 (0.04)	2.38 (1.42)
2K	bpEQ	0.23 (0.14)	1.19 (0.06)
	bpMAF	0.28 (0.11)	1.19 (0.30)
	lduMAF	0.46 (0.04)	1.19 (1.19)
3K	bpEQ	0.23 (0.14)	0.79 (0.06)
	bpMAF	0.30 (0.12)	0.79 (0.23)
	lduMAF	0.46 (0.03)	0.79 (0.92)
6K	bpEQ	0.23 (0.14)	0.39 (0.07)
	bpMAF	0.29 (0.11)	0.39 (0.17)
	lduMAF	0.43 (0.05)	0.39 (0.63)

Properties of low density panel SNPs for ECA1 selected using three methods, as calculated in the within-population analysis of the UK dataset; [1]total number of SNPs that would be on a genome-wide LDP of equivalent density.

b) ECA10

**Number of SNPs**[I]	**Method**	**Mean (SD) MAF**	**Mean (SD) distance between SNPs (Mb)**
384	bpEQ	0.25 (0.16)	6.46 (0.11)
	bpMAF	0.29 (0.14)	6.46 (0.17)
	lduMAF	0.43 (0.10)	6.45 (2.51)
768	bpEQ	0.22 (0.14)	3.23 (0.08)
	bpMAF	0.25 (0.12)	3.23 (0.30)
	lduMAF	0.44 (0.07)	3.23 (2.16)
1K	bpEQ	0.26 (0.16)	2.47 (0.06)
	bpMAF	0.33 (0.12)	2.47 (0.28)
	lduMAF	0.44 (0.07)	2.47 (1.89)
2K	bpEQ	0.24 (0.14)	1.20 (0.06)
	bpMAF	0.29 (0.11)	1.20 (0.20)
	lduMAF	0.46 (0.05)	1.20 (0.95)
3K	bpEQ	0.25 (0.13)	0.80 (0.06)
	bpMAF	0.30 (0.12)	0.80 (0.16)
	lduMAF	0.47 (0.04)	0.80 (0.75)
6K	bpEQ	0.26 (0.14)	0.40 (0.07)
	bpMAF	0.31 (0.11)	0.40 (0.10)
	lduMAF	0.46 (0.04)	0.40 (0.44)

Properties of low density panel SNPs for ECA10 selected using three methods, as calculated in the within-population analysis of the UK dataset; [1]total number of SNPs that would be on a genome-wide LDP of equivalent density.

c) ECA20

**Number of SNPs**[I]	**Method**	**Mean (SD) MAF**	**Mean (SD) distance between SNPs (Mb)**
384	bpEQ	0.28 (0.13)	7.06 (0.21)
	bpMAF	0.33 (0.12)	7.06 (0.09)
	lduMAF	0.44 (0.09)	7.06 (2.20)
768	bpEQ	0.26 (0.15)	3.18 (0.16)
	bpMAF	0.29 (0.11)	3.18 (0.12)
	lduMAF	0.46 (0.06)	3.18 (1.25)
1K	bpEQ	0.21 (0.13)	2.44 (0.14)
	bpMAF	0.40 (0.08)	2.44 (0.34)
	lduMAF	0.46 (0.05)	2.44 (1.12)
2K	bpEQ	0.24 (0.14)	1.20 (0.11)
	bpMAF	0.29 (0.11)	1.20 (0.18)
	lduMAF	0.46 (0.04)	1.20 (0.62)
3K	bpEQ	0.26 (0.14)	0.79 (0.10)
	bpMAF	0.30 (0.11)	0.79 (0.14)
	lduMAF	0.47 (0.04)	0.79 (0.53)
6K	bpEQ	0.25 (0.14)	0.40 (0.06)
	bpMAF	0.29 (0.11)	0.39 (0.10)
	lduMAF	0.46 (0.03)	0.39 (0.41)

Properties of low density panel SNPs for ECA20 selected using three methods, as calculated in the within-population analysis of the UK dataset; [1]total number of SNPs that would be on a genome-wide LDP of equivalent density.

d) ECA26

**Number of SNPs[I]**	**Method**	**Mean (SD) MAF**	**Mean (SD) distance between SNPs (Mb)**
384	bpEQ	0.28 (0.16)	6.89 (0.16)
	bpMAF	0.30 (0.13)	6.89 (0.05)
	lduMAF	0.47 (0.02)	6.89 (1.99)
768	bpEQ	0.27 (0.15)	3.18 (0.14)
	bpMAF	0.36 (0.12)	3.18 (0.17)
	lduMAF	0.47 (0.02)	3.18 (1.34)
1K	bpEQ	0.27 (0.15)	2.43 (0.16)
	bpMAF	0.45 (0.03)	2.43 (0.23)
	lduMAF	0.47 (0.05)	2.42 (1.39)
2K	bpEQ	0.28 (0.16)	1.22 (0.12)
	bpMAF	0.34 (0.13)	1.22 (0.13)
	lduMAF	0.47 (0.02)	1.22 (0.61)
3K	bpEQ	0.30 (0.14)	0.79 (0.08)
	bpMAF	0.33 (0.13)	0.79 (0.12)
	lduMAF	0.47 (0.02)	0.79 (0.59)
6K	bpEQ	0.28 (0.15)	0.40 (0.07)
	bpMAF	0.31 (0.12)	0.39 (0.09)
	lduMAF	0.47 (0.02)	0.39 (0.40)

Properties of low density panel SNPs for ECA26 selected using three methods, as calculated in the within-population analysis of the UK dataset; [1]total number of SNPs that would be on a genome-wide LDP of equivalent density.
