# Supplementary material for: The utility of low-density genotyping for imputation in the Thoroughbred horse
Source: Genet Sel Evol. 2014 Feb 4;46(1):9. doi: 10.1186/1297-9686-46-9 (PMC3930001; doi:10.1186/1297-9686-46-9)
Supplement: Additional file 7: Table S4 — The mean correlation between true and predicted genotypes, as calculated in the within-population analysis of the UK dataset. The data provided represent the results of imputation from LDPs with SNPs selected by Methods 1 to 3, expressed as the mean correlation between true and predicted genotypes, both per individual and per SNP. Results are shown for chromosomes 1, 10, 20 and 26. [file 1297-9686-46-9-S7.pdf]

Table S4 The mean correlation between true and predicted genotypes, as calculated in the within-population analysis of the UK dataset

a) Per individual

Chromosome	Number of SNPs[1]	bpEQ	bpMAF	lduMAF
ECA1	384	0.46 (0.14,0.89)	0.49 (0.20,0.91)	0.53 (0.22,0.89)
	768	0.64 (0.36,0.93)	0.66 (0.38,0.93)	0.69 (0.37,0.94)
	1K	0.70 (0.41,0.93)	0.78 (0.51,0.96)	0.75 (0.47,0.98)
	2K	0.86 (0.53,0.99)	0.88 (0.62,0.98)	0.85 (0.52,0.99)
	3K	0.92 (0.59,0.99)	0.94 (0.60,1.00)	0.88 (0.48,0.99)
	6K	0.97 (0.73,1.00)	0.97 (0.71,1.00)	0.93 (0.61,1.00)
ECA10	384	0.46 (0.11,0.98)	0.43 (0.04,0.98)	0.42 (0.00,0.94)
	768	0.58 (0.20,0.97)	0.57 (0.04,0.98)	0.59 (0.26,0.99)
	1K	0.67 (0.32,0.98)	0.68 (0.29,0.97)	0.66 (0.14,0.99)
	2K	0.82 (0.56,0.98)	0.85 (0.59,0.98)	0.86 (0.42,1.00)
	3K	0.89 (0.70,1.00)	0.91 (0.76,1.00)	0.91 (0.62,1.00)
	6K	0.96 (0.80.1.00)	0.97 (0.87,1.00)	0.95 (0.76,1.00)
ECA20	384	0.37 (0.07,0.89)	0.36 (-0.01,0.83)	0.37 (-0.06,0.84)
	768	0.51 (0.13,0.93)	0.53 (0.09,0.92)	0.54 (0.15,0.91)
	1K	0.55 (0.12,0.93)	0.63 (0.27,0.99)	0.62 (0.24,0.97)
	2K	0.76 (0.30,0.98)	0.80 (0.42,0.99)	0.82 (0.52,0.99)
	3K	0.87 (0.58,0.99)	0.89 (0.63,0.99)	0.89 (0.46,1.00)
	6K	0.95 (0.79,1.00)	0.96 (0.85,1.00)	0.93 (0.72,1.00)
ECA26	384	0.38 (-0.04,0.94)	0.40 (0.01,0.93)	0.44 (-0.02,0.98)
	768	0.57 (0.17,0.97)	0.60 (0.16,0.94)	0.61 (0.13,0.97)
	1K	0.60 (0.14, 0.95)	0.67 (0.25,0.98)	0.70 (0.30,1.00)
	2K	0.76 (0.29,1.00)	0.81 (0.31,1.00)	0.84 (0.16,1.00)
	3K	0.90 (0.36,1.00)	0.90 (0.62,1.00)	0.90 (0.54,1.00)
	6K	0.96 (0.78,1.00)	0.96 (0.79,1.00)	0.91 (0.49,1.00)

Mean correlation between true and predicted genotypes per individual, with minimum and maximum values in brackets; [1]total number of SNPs that would be on a genome-wide LDP of equivalent density.

b) Per SNP

Chromosome	Number of SNPs[1]	bpEQ	bpMAF	lduMAF
ECA1	384	0.30 (-0.17,1.00)	0.32 (-0.14,1.00)	0.36 (-0.08,1.00)
	768	0.52 (-0.08,1.00)	0.53 (-0.06,1.00)	0.55 (-0.05,1.00)
	1K	0.60 (-0.04,1.00)	0.67 (-0.05,1.00)	0.64 (-0.05,1.00)
	2K	0.81 (-0.04,1.00)	0.83 (-0.02,1.00)	0.79 (-0.02,1.00)
	3K	0.89 (-0.01,1.00)	0.90 (-0.03,1.00)	0.83 (-0.02,1.00)
	6K	0.95 (0.25,1.00)	0.96 (0.49,1.00)	0.90 (-0.01,1.00)
ECA10	384	0.31 (-0.13,1.00)	0.28 (-0.14,1.00)	0.26 (-0.09,1.00)
	768	0.46 (-0.10,1.00)	0.45 (-0.09,1.00)	0.46 (-0.08,1.00)
	1K	0.59 (-0.06,1.00)	0.58 (-0.08,1.00)	0.55 (-0.04,1.00)
	2K	0.76 (-0.04,1.00)	0.79 (-0.06,1.00)	0.80 (-0.02,1.00)
	3K	0.85 (0.20,1.00)	0.88 (-0.01,1.00)	0.87 (0.13, 1.00)
	6K	0.94 (0.34,1.00)	0.95 (0.34,1.00)	0.93 (-0.01, 1.00)
ECA20	384	0.24 (-0.16,1.00)	0.22 (-0.15,1.00)	0.23 (-0.15,1.00)
	768	0.40 (-0.08,1.00)	0.41 (-0.06,1.00)	0.43 (-0.04,1.00)
	1K	0.46 (-0.06,1.00)	0.54 (-0.03,1.00)	0.52 (-0.03,1.00)
	2K	0.69 (-0.06,1.00)	0.75 (-0.02,1.00)	0.76 (-0.04,1.00)
	3K	0.83 (0.06,1.00)	0.86 (-0.03,1.00)	0.86 (0.17,1.00)
	6K	0.93 (0.34,1.00)	0.94 (0.23,1.00)	0.92 (0.40,1.00)
ECA26	384	0.20 (-0.17,1.00)	0.23 (-0.16,1.00)	0.27 (-0.13,1.00)
	768	0.44 (-0.03,1.00)	0.46 (-0.04,1.00)	0.47 (-0.03,1.00)
	1K	0.48 (-0.04,1.00)	0.54 (-0.03,1.00)	0.56 (-0.06,1.00)
	2K	0.67 (-0.03,1.00)	0.74 (-0.02,1.00)	0.76 (-0.04,1.00)
	3K	0.86 (-0.03,1.00)	0.85 (-0.01,1.00)	0.85 (0.21,1.00)
	6K	0.94 (0.50,1.00)	0.95 (0.69,1.00)	0.88 (-0.01,1.00)

Mean correlation between true and predicted genotypes per SNP, with minimum and maximum values in brackets; [1]total number of SNPs that would be on a genome-wide LDP of equivalent density.
